# Sensory Characteristics and Consumer Acceptance of Commercial Yakgwa, a Traditional Korean Dessert, Among Vietnamese Consumers: A Check-All-That-Apply (CATA) Approach

**DOI:** 10.3390/foods15203654

**Published:** 2026-10-15

**Authors:** Juyoun Lee

**Affiliations:** Department of Food and Nutrition, Duksung Women’s University, 33 Samyang-ro, Seoul 01369, Republic of Korea; foodinstitute@hanmail.net

**Keywords:** yakgwa, check-all-that-apply (CATA), sensory evaluation, traditional Korean dessert

## Abstract

To explore the overseas potential of yakgwa, a traditional Korean honey confection, this study investigated the sensory characteristics and consumer acceptability of commercial yakgwa among Vietnamese consumers. Four products with distinct sensory characteristics were selected from 12 commercial products. A total of 104 Vietnamese consumers evaluated the samples using a check-all-that-apply (CATA) questionnaire comprising 39 sensory and image attributes, and rated acceptability, familiarity, willingness to eat again, and recommendation intention on 9-point scales. Data were analyzed using Cochran’s Q test, analysis of variance, correspondence analysis, and multiple factor analysis (MFA). Samples 537 and 681 received significantly higher ratings than samples 214 and 825 for overall and most other acceptability ratings, familiarity, willingness to eat again, and recommendation intention. In the CATA evaluation, sample 214 was frequently described as oily-looking, crispy, and dry and rough, whereas sample 825 was associated with dark-colored, salty taste, herbal flavor, and cinnamon and ginger attributes. Samples 537 and 681 were more frequently described as light-colored, sweet-tasting, and soft. In the MFA, samples 537 and 681 were positioned closer to the acceptability and behavioral intention attributes than samples 214 and 825. Thus, light color, sweetness, and soft texture may be associated with higher acceptability, whereas oily appearance, dry and rough texture, and strong spice flavors may be associated with lower acceptability. However, this study was conducted in a single region and included many consumers unfamiliar with yakgwa; further research covering broader regions and age groups is needed.

## 1. Introduction

Traditional foods are cultural assets that embody the history, dietary habits, and cultural identity of a nation or region. Consumers associate traditional foods with region-specific ingredients and production methods, as well as with intergenerational transmission and unique sensory characteristics [1,2]. Traditional foods must therefore continue to evolve in response to changing consumer demands and market conditions while preserving their cultural identity. Product innovation that enhances safety, health benefits, convenience, and sensory quality without compromising tradition has been identified as a key factor in strengthening the sustainability and market competitiveness of traditional foods [1,3].

Entering overseas markets requires traditional foods to account for the cultural background and dietary experiences of target consumers. Because familiar tastes, aromas, and textures vary across cultures, the same food can be perceived and evaluated differently by different consumer groups [4,5]. In particular, the acceptability of unfamiliar foods is influenced by their similarity to previously experienced foods, repeated exposure, and familiarity [6]. A study on Malaysian consumers found that prior experience with and familiarity toward Korean food had a greater influence on the acceptability of Korean traditional foods than the provision of simple product information [7]. Yakgwa, one of Korea’s representative traditional foods, is a confectionery made by kneading wheat flour dough with sesame oil and honey or grain syrup, deep-frying it, and soaking it in syrup. The name yakgwa combines yak (medicine) and gwa (confection), literally meaning “medicinal confection,” and reflects the traditional view of the confection as a precious food made with ingredients considered beneficial to health, such as sesame oil and honey [2]. It has long been used in holidays, ancestral rites, and various ceremonial occasions, and is widely recognized as a food that represents Korean dietary culture and tradition [2].

Such cultural differences have also been observed for yakgwa. A study evaluating yakgwa among Korean, Japanese, and French consumers found that Korean consumers preferred a soft, cohesive texture, whereas Japanese and French consumers showed a relatively stronger preference for a layered, crispy texture [8]. Similar cross-cultural differences have been reported for other Korean traditional foods; for example, Chinese consumers rated ready-to-heat bulgogi as excessively sweet, whereas Korean consumers considered the same sweetness level appropriate [9,10]. Higher familiarity with a food has also been reported to increase sensory liking [11]. Similarly, a study on traditional Japanese confectionery found that familiar aromas enhanced flavor liking, whereas this effect was not observed for unfamiliar foods [12]. These findings indicate that both cultural experience and sensory characteristics should be considered together when evaluating traditional foods among overseas consumers.

The appearance, color, and aroma of a food shape sensory expectations prior to consumption, which can in turn influence actual sensory perception and liking [13]. Understanding the relationship between such expectations and actual perception therefore requires analyzing not only overall liking but also the specific sensory attributes perceived by consumers. Consumer-based sensory evaluation has been widely used in recent years to efficiently capture this relationship [14,15]. Check-All-That-Apply (CATA) is a method in which consumers select, from a list of provided attributes, all terms they consider applicable to describe a product after tasting it [16]. CATA allows untrained consumers to participate easily and enables sensory characterization based on consumers’ own language. It can also be used to compare sensory differences among products and to visually analyze the relationship between sensory attributes and consumer liking [15,16,17]. Because the composition and number of terms used in CATA can influence consumer responses and the resulting product characterization, selecting terms that are easy to understand and capable of distinguishing between products is essential [18].

Previous studies on yakgwa have mainly focused on functional ingredients, manufacturing methods, physicochemical quality, and storage stability [19,20,21,22,23]. Meanwhile, a study evaluating 12 commercial yakgwa products found that textural differences arising from manufacturing type served as a key sensory criterion distinguishing the products [24], highlighting the need to examine sensory differences among commercial yakgwa products from the consumer’s perspective.

Vietnam is one of the markets showing strong interest in Korean cultural content and food. According to a survey on the global status of the Korean Wave, 82.9% of Vietnamese consumers reported a favorable attitude toward Korean cultural content. Korean food ranked second only to K-pop among images associated with Korea, and food accounted for 64.7% of preferred Korean products and services [25]. Reflecting this trend, a recent study reported that favorability toward Korean culture positively influences Vietnamese consumers’ trust in and purchase intention toward Korean food products [26]. These findings underscore the need to examine the sensory acceptability of Korean traditional foods among Vietnamese consumers. For cultural interest to translate into actual purchase and continued consumption, it is necessary to identify the specific tastes, aromas, colors, and textures preferred by Vietnamese consumers.

To date, no study has examined the relationship between the sensory characteristics of commercial yakgwa and consumer liking among Vietnamese consumers. Accordingly, this study used the CATA method to analyze the sensory characteristics and consumer liking of four commercial Korean yakgwa products as perceived by Vietnamese consumers. Correspondence analysis (CA) and multiple factor analysis (MFA) were further applied to examine the relationship between sensory differences among samples and consumer responses. This study aimed to suggest directions for product differentiation and sensory quality improvement of yakgwa for Vietnamese consumers and to provide foundational data for expanding the overseas market for K-dessert products.

## 2. Materials and Methods

### 2.1. Selection of Yakgwa Samples

**Figure 1:**
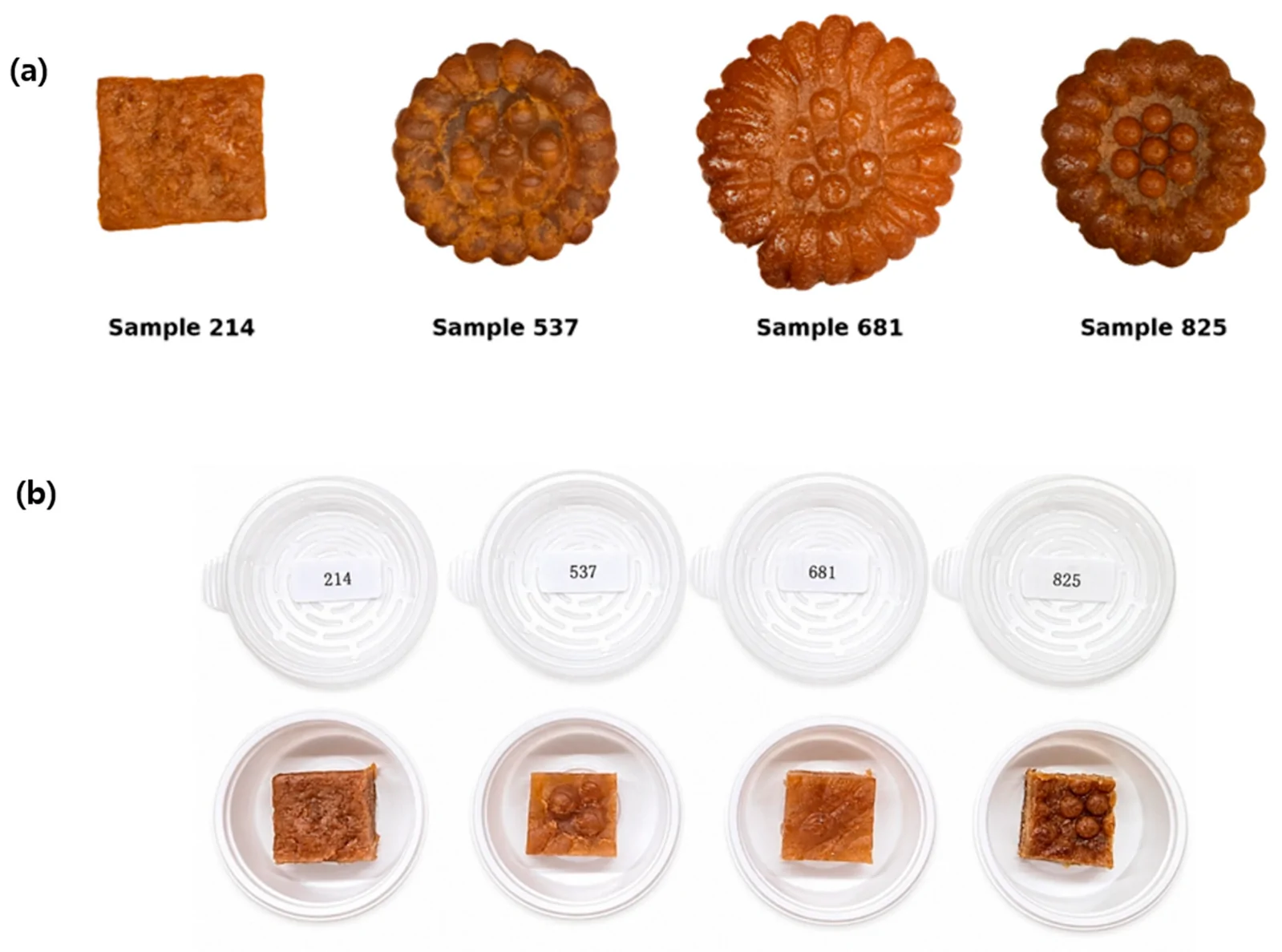
Appearance of the four commercial yakgwa samples and their presentation for consumer evaluation. (**a**) Whole samples labeled with three-digit random codes. (**b**) Samples served in coded containers during the evaluation session.

To reflect the diversity of yakgwa products currently available on the Korean market, this study surveyed products sold through domestic online shopping malls, large supermarkets, and specialty confectionery brands, and purchased a total of 12 preliminary samples. These samples represented diverse manufacturing methods and product characteristics, including mass-produced products, handmade products manufactured and sold by small-scale independent producers, fusion products that reinterpret traditional yakgwa in a modern style, and products made using a layered-structure manufacturing technique. In CATA studies, it is important to select samples with sufficiently distinguishable sensory characteristics [27]. Accordingly, one product was selected from each of these four types, which were expected to differ in sensory characteristics because of their different manufacturing methods. Within each type, selection was based on product popularity, as indicated by online consumer reviews, and availability for online purchase. The four selected products were used in the consumer acceptability and CATA evaluations. Following selection, these products were evaluated by 13 trained panelists with experience in the sensory evaluation of yakgwa to refine the CATA terms, as described in Section 2.3. The product categories and ingredient lists are presented in Table 1. Samples were purchased separately for each evaluation session. For both purchase batches, the samples were cut into serving-size portions (2 × 2 cm) immediately after purchase, placed in lidded containers, and stored at 4 °C (Figure 1). The samples were evaluated within 9 days of purchase in Session 1 and within 4 days of purchase in Session 2. One hour before each evaluation session, the samples were removed from refrigeration and held at room temperature (26 °C) until serving. All evaluations were conducted within the labeled shelf life of each product. The purchase dates, evaluation session periods, and the manufacturing or use-by dates indicated on the product packaging are presented in Table 2, and the nutrition information declared on the product labels is provided in Table 3.

### 2.2. Participants and Experimental Design

The consumer evaluation was conducted in two sessions at Duksung Women’s University in Seoul: Session 1 from 2 July to 10 July 2026, and Session 2 from 11 to 14 September 2026. The survey was administered to 104 Vietnamese consumers who voluntarily agreed to participate after receiving an explanation of the study’s objectives and evaluation methods. In the same session, both a Check-All-That-Apply (CATA) evaluation and a consumer acceptability evaluation were conducted for each sample. To prevent bias from product information, the evaluation samples were labeled with three-digit random numbers (214, 537, 681, 825). Additionally, to minimize the influence of presentation order, four different presentation sequences (214-537-681-825, 537-681-825-214, 681-825-214-537, 825-214-537-681) were established, and participants were evenly assigned to each sequence.

### 2.3. Check-All-That-Apply (CATA) Evaluation

The sensory attributes and image-related attribute categories used in the CATA were selected to form primary attributes based on prior research on yakgwa [28,29,30,31]. Subsequently, additional CATA terms were collected using a free-listing method, in which consumers were asked to freely describe the characteristics they perceived while consuming the product [32]. To ensure that the terms reflected the perceptions of the target consumers as well as those of consumers familiar with yakgwa, 50 university students, comprising 28 Vietnamese and 22 Korean students, were presented with commercially available yakgwa and asked to freely describe its sensory characteristics, such as appearance, taste, and aroma. A total of 386 terms were collected through the free-listing method, and after excluding duplicates, 87 terms were derived. Terms that differed in expression but were similar in meaning were consolidated into terms that were easier for consumers to understand. Expressions with unclear meanings or low relevance to the sensory characteristics of yakgwa were excluded. Through this refinement process, a list of 50 candidate terms was compiled. The 13 trained panelists described in Section 2.1 then evaluated the four selected samples and reviewed the candidate terms for comprehensibility, overlap in sensory meaning, and ability to distinguish between samples, identifying inaccurate or confusing terms and suggesting additional terms. As a result, 39 final CATA terms were selected. The questionnaire, including the CATA terms, was presented in Korean and English. A Vietnamese version was not prepared. Instead, Vietnamese assistants fluent in Korean were present during the evaluation sessions and provided explanations in Vietnamese when participants had difficulty understanding the questionnaire or individual CATA terms, to support participants’ understanding.

The final selected sensory attribute terms included 5 terms for Appearance (light-colored, dark-colored, glossy, oily-looking, dry-looking), 7 for Aroma (honey aroma, rice syrup aroma, ginger aroma, cinnamon aroma, nutty aroma, stale oily odor, weak aroma), 12 for Taste/Flavor (sweet taste, excessively sweet taste, honey flavor, nutty flavor, rice syrup flavor, cinnamon flavor, oily flavor, salty taste, rich flavor, herbal flavor, ginger flavor, wheat flour flavor), and 10 for Texture (crispy, soggy, hard, soft, moist, chewy, dense, sticks to teeth, dry and rough, light mouthfeel). Image category consisted of 5 terms (traditional snack-like, trendy, premium dessert-like, gift-worthy, familiar), for a total of 39 items. Water was provided between samples to minimize carryover effects.

This study was conducted with the approval of the Institutional Review Board of Duksung Women’s University (IRB Approval No.: 2026-006-015-A).

### 2.4. Consumer Acceptance Evaluation

The consumer acceptability evaluation was conducted using a self-reporting method, in which questionnaires and samples were distributed for participants to complete. To prevent order-related bias, participants were assigned random numbers and divided into four groups, with the order of sample presentation varied among the groups. The evaluation criteria included appearance, color, aroma, sweetness, nutty flavor, oiliness, texture, aftertaste, flavor, and overall acceptability. Recommendation intention and familiarity were composed of willingness to eat again, recommendation intention, and familiarity, and were evaluated using a 9-point scale (1 = dislike extremely, 5 = neither like nor dislike, 9 = like extremely).

### 2.5. Statistical Analysis

General characteristics were expressed as frequencies and percentages, while consumer preference, willingness to eat again, willingness to recommend, and familiarity were presented as means and standard deviations (mean ± standard deviation). To identify significant differences among samples, a one-way analysis of variance (ANOVA) was conducted, followed by Duncan’s multiple range test for post-hoc analysis when significant differences were found. For the CATA data, the selection frequency and percentage for each attribute were calculated, and Cochran’s Q test was performed to analyze differences in sensory attributes among samples. Additionally, correspondence analysis (CA) was conducted to visually examine the relationship between samples and sensory attributes. To comprehensively analyze the relationship between CATA attributes and consumer preference, multiple factor analysis (MFA) was performed, which confirmed the relative positions of the samples and the relationships among CATA attributes and consumer preference variables. SPSS Statistics 26.0 (IBM Corp., Armonk, NY, USA) was used for the analysis of general characteristics and consumer preference, while XLSTAT software (Version 2026.01, Addinsoft, Paris, France) was used for CATA, Correspondence Analysis, and MFA.

## 3. Results and Discussion

### 3.1. Characteristics of Yakgwa Consumption Among Study Participants

The study participants’ yakgwa consumption history is shown in Table 4. All 104 participants were women aged 18–29 years, with a mean age of 21.85 ± 2.55 years. Of the 104 participants, 62 (59.6%) had experience consuming yakgwa, a proportion similar to that of first-time consumers. Most of those with consumption experience reported eating yakgwa rarely or only 1–2 times a year, indicating that the overall frequency of yakgwa consumption was low. Among those with consumption experience, 37 participants (59.7%) had purchased yakgwa. The average preference score for yakgwa was 3.24 (5-point scale; 1 = do not like at all, 3 = neutral, 5 = like very much), and the average level of interest in traditional Korean desserts was 3.62 (5-point scale; 1 = not at all interested, 3 = neutral, 5 = very interested), which was slightly higher than the preference score for yakgwa.

Table 5 shows the primary reasons for consuming or purchasing yakgwa and the factors considered when making a purchase. Among the 68 respondents, the most frequently cited reasons for consumption were as a snack (48.5%) and to experience traditional food (47.1%). The most important factor considered when purchasing was taste (82.4%), followed by price (29.4%), texture (23.5%), and healthiness (22.1%).

### 3.2. Consumer Acceptability of Commercial Yakgwa Samples

Table 6 presents Vietnamese consumers’ acceptability ratings for four commercially available yakgwa samples, along with their familiarity ratings, willingness to eat again, and recommendation intention. Significant differences among the samples were observed for all evaluated measures except oiliness (*p* < 0.05).

Samples 537 and 681 received significantly higher appearance ratings than samples 214 and 825 (*p* < 0.001). Sample 825 received the lowest color rating, significantly lower than those of the other three samples (*p* < 0.001), and its aroma rating was significantly lower than those of samples 537 and 681 (*p* < 0.05). Samples 537 and 681 received significantly higher ratings than samples 214 and 825 for sweetness, texture, aftertaste, flavor, and overall acceptability (*p* < 0.05), as well as for willingness to eat again, recommendation intention, and familiarity (*p* < 0.001). Nutty flavor ratings were significantly higher for samples 537 and 681 than for sample 825 (*p* < 0.05), while sample 214 had an intermediate rating. Oiliness ratings did not differ significantly among the samples (*p* = 0.071). Overall, samples 537 and 681 were evaluated more favorably by Vietnamese consumers. Previous research has reported a positive association between food familiarity and sensory acceptability [11]. In a study involving Korean, Japanese, and French consumers, familiarity with yakgwa was correlated with all hedonic ratings among Korean consumers, who were most familiar with the product, but with only some ratings among Japanese and French consumers [8]. Familiarity with Asian foods has also been reported to influence purchase intention, while familiar tastes, appearances, and cooking methods have been suggested as ways to facilitate the acceptance of novel foods [33]. In addition, food neophobia has been identified as a challenge in introducing traditional foods to foreign markets, whereas greater familiarity has been associated with higher overall acceptability [7].

In the present study, samples 537 and 681 received relatively high ratings for both familiarity and overall acceptability. This pattern is consistent with previous findings, although the association between these two measures was not directly tested.

### 3.3. CATA Results for Commercial Yakgwa Samples

Table 7 summarizes the sensory characteristics of yakgwa evaluated using 39 sensory attributes and the CATA method. This study evaluated the sensory characteristics of four commercially available yakgwa products among 104 Vietnamese consumers. For appearance, samples 537 and 681 were most frequently described as light-colored (59.62% and 61.54%, respectively), whereas sample 825 was predominantly characterized as dark-colored (80.77%). Sample 214 showed a significantly higher selection frequency for oily-looking (45.19%) than the other samples. In the aroma evaluation, sample 537 showed the highest selection frequencies for honey aroma and nutty aroma (34.62% each), while it was also most frequently associated with weak aroma (28.85%). Ginger aroma was selected significantly more frequently for sample 825 (17.31%) than for sample 537 (3.85%), whereas samples 214 and 681 did not differ significantly from the other samples.

Taste and flavor results showed that sample 537 was most frequently described as having a sweet taste (53.85%). Sample 214 showed relatively high selection frequencies for nutty flavor (35.58%) and oily flavor (38.46%), whereas sample 825 was characterized by relatively high frequencies of excessively sweet taste (16.35%), salty taste (24.04%), and herbal flavor (20.19%). In particular, salty taste and herbal flavor were selected significantly more frequently for sample 825 than for the other samples. Texture results indicated that sample 214 showed significantly higher selection frequencies for crispy (23.08%) and dry and rough (33.65%) than the other samples, whereas soft was selected significantly less frequently for sample 214 (26.92%). A traditional snack-like image was selected at relatively high frequencies for all four samples (52.88–62.50%), with no significant differences among the samples, including the fusion-style sample 825, indicating that the participants generally perceived yakgwa as a food strongly associated with tradition regardless of sensory differences. This consistent traditional image may help differentiate yakgwa from other sweet snacks, and the result for the fusion-style sample suggests that modern reinterpretation may be possible without compromising its traditional character [1,3]. However, further research is needed to determine whether this image contributes to consumer acceptance in the Vietnamese market.

Overall, the differences in selection frequencies indicate that Vietnamese consumers perceived distinct sensory profiles among the four yakgwa products. Sensory characteristics such as color and aroma shape expectations before consumption and may influence subsequent sensory evaluations and product preferences [13]. Furthermore, consumers’ evaluations of unfamiliar foods may vary according to their cultural backgrounds and familiarity with the foods or similar products [8,11].

### 3.4. Correspondence Analysis of CATA Attributes and Yakgwa Samples

**Figure 2:**
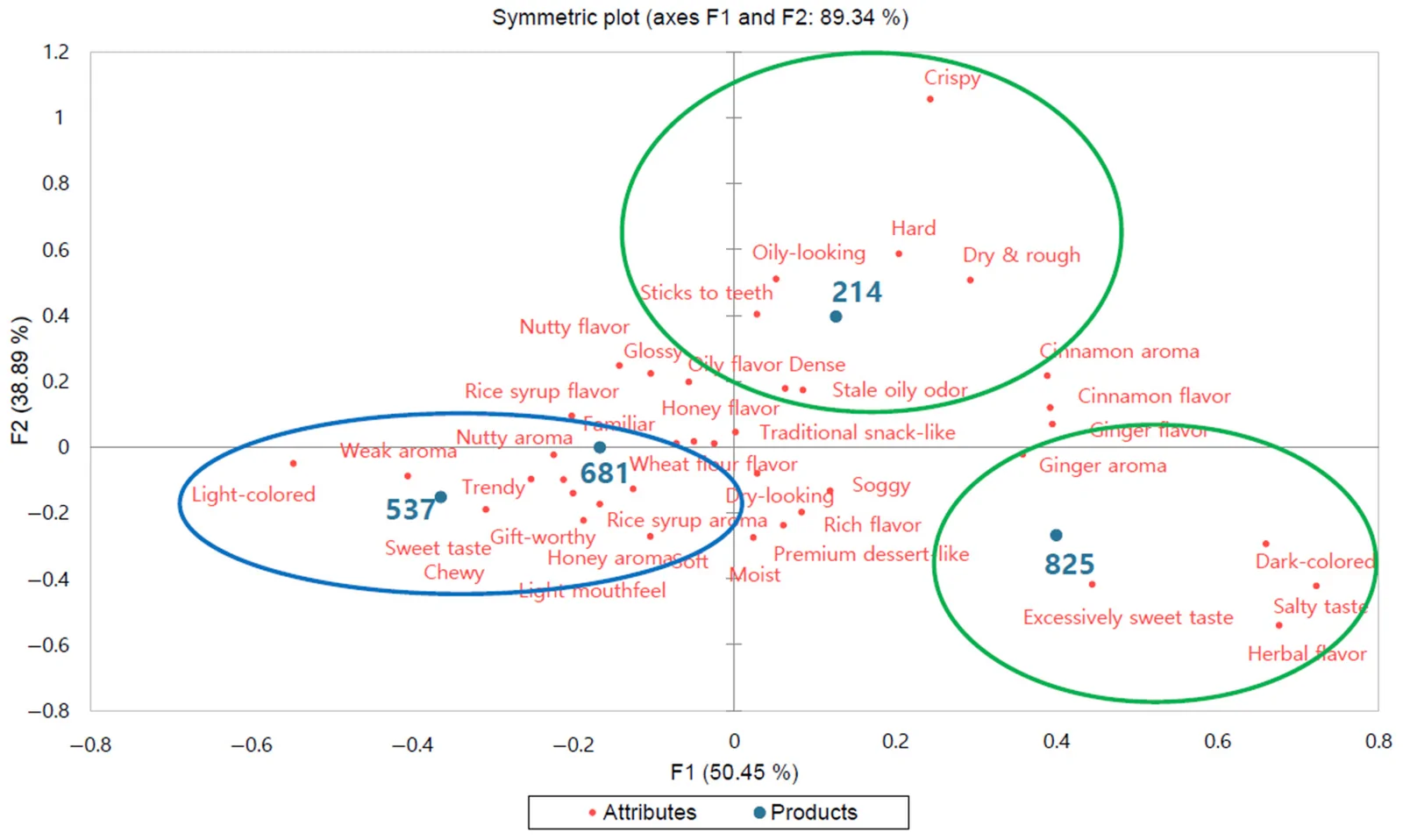
Correspondence analysis biplot of CATA attributes for the four commercial yakgwa samples evaluated by Vietnamese consumers. F1 and F2 explained 50.45% and 38.89% of the total inertia, respectively, together accounting for 89.34%. Blue ellipses indicate samples with relatively higher mean overall acceptability (537 and 681), whereas green ellipses indicate those with relatively lower mean overall acceptability (214 and 825). The ellipses were drawn manually as visual guides only and do not represent statistical confidence regions.

Figure 2 shows the results of correspondence analysis (CA) using the CATA sensory attributes. The first dimension (F1) explained 50.45% of the total inertia, and the second dimension (F2) explained 38.89%, resulting in a cumulative proportion of explained inertia of 89.34%. Sample 214 was positioned close to crispy, hard, dry and rough, oily-looking, and sticks to teeth. Sample 537 was associated with light-colored, weak aroma, and sweet taste, and was also located relatively close to soft, chewy, honey aroma, and light mouthfeel. Sample 681 was positioned near familiar, wheat flour flavor, honey flavor, nutty aroma, and rice syrup aroma. Sample 825 was located close to dark-colored, salty taste, herbal flavor, and excessively sweet taste, with ginger aroma, cinnamon aroma, ginger flavor, and cinnamon flavor oriented in the same direction. Notably, along the second dimension, crispy and hard were positioned opposite to soft and chewy, suggesting that texture was a key sensory dimension differentiating the commercially available yakgwa products.

Previous research has reported that textural differences among commercially available yakgwa products, arising from variations in manufacturing methods, contribute to consumer differentiation among products [24], which is consistent with the present findings. In addition, responses to the tastes and aromas of traditional foods have been reported to vary according to consumers’ cultural experiences and familiarity with the food [5,12]. The differences in cinnamon aroma, ginger aroma, rice syrup flavor, nutty flavor, and texture identified in this study may therefore be relevant to Vietnamese consumers’ acceptance of yakgwa. Taken together, these results suggest that texture should be considered alongside taste and aroma in the development of yakgwa products for the Vietnamese market.

### 3.5. Relationship Between CATA Sensory Characteristics and Consumer Acceptability

**Figure 3:**
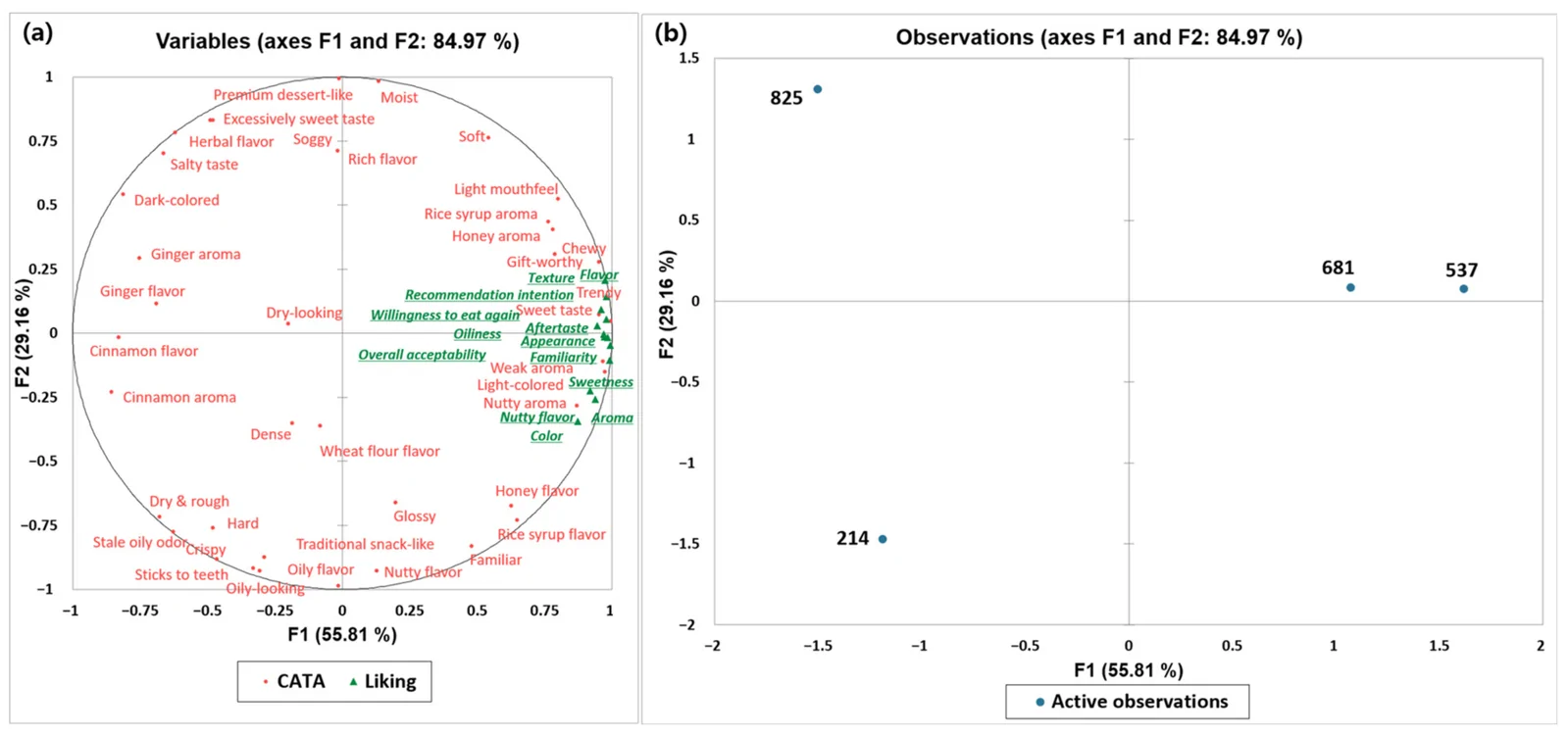
Multiple Factor Analysis (MFA) of the four commercial yakgwa samples. (**a**) Relationships among CATA sensory attributes and consumer acceptability and behavioral intention variables. (**b**) Distribution of the samples. F1 and F2 explained 55.81% and 29.16% of the total variance, respectively, together accounting for 84.97%.

Multiple factor analysis (MFA) was conducted to examine the relationship between the sensory characteristics obtained from the CATA questions and the acceptability responses of the four yakgwa samples. The results showed that the first dimension (F1) explained 55.81% and the second dimension (F2) explained 29.16% of the total variance, with a cumulative value of 84.97% (Figure 3). All acceptability attributes (overall acceptability, appearance, color, aroma, flavor, texture, sweetness, oiliness, aftertaste, familiarity, willingness to eat again, and recommendation intention) were clustered near the positive direction of the first dimension (F1), indicating that the acceptability responses were highly intercorrelated and were primarily explained by F1. The positive direction of F1 was associated with sweet taste, honey aroma, rice syrup aroma, nutty aroma, light-colored, soft, chewy, light mouthfeel, and familiar, whereas the negative direction was associated with dark-colored, salty taste, herbal flavor, ginger aroma, ginger flavor, cinnamon aroma, cinnamon flavor, hard, crispy, dry and rough, sticks to teeth, and stale oily odor. The second dimension (F2) was primarily related to texture- and moisture-related attributes, with moist, soft, and soggy located in the positive direction and crispy, hard, dry and rough, and oily-looking located in the negative direction. In the observation plot, samples 537 and 681 were located on the positive side of F1, close to the acceptability attributes, whereas samples 825 and 214 were positioned on the negative side. Sample 537 was associated with nutty aroma, nutty flavor, rice syrup flavor, weak aroma, and light color, and was located in a similar direction to the acceptability attributes of aroma, color, overall acceptability, familiarity, and recommendation intention. Sample 681 was associated with honey aroma, honey flavor, and a gift-worthy image, and was located close to the acceptability attributes of taste, texture, appearance, and aftertaste. Willingness to eat again and sweetness were located near the center of the positive side of F1 and therefore could not be clearly attributed to either sample 537 or sample 681. Samples 825 and 214 were further separated along F2. Sample 825 was located in the upper-left quadrant, in the direction of dark-colored, salty taste, herbal flavor, ginger aroma, ginger flavor, cinnamon aroma, cinnamon flavor, excessively sweet taste, and moist, together with a premium dessert-like image. Sample 214 was located in the lower-left quadrant, in the direction of crispy, hard, dry and rough, sticks to teeth, oily-looking, oily flavor, and stale oily odor. These results indicate that the two samples positioned away from the acceptability attributes were characterized by different sensory profiles: sample 825 by salty taste, herbal flavor, and spice-related attributes accompanied by excessively sweet taste, and sample 214 by a hard, dry, and oily character. Consumer responses to spice-related flavors have been reported to depend on prior experience and familiarity with the flavors. Lee et al. [34] reported that Korean and US consumers perceived hot sauces containing gochujang differently, and that this difference appeared to originate from differences in flavor familiarity. Similarly, Kim et al. [35] found that the drivers of liking for sauces made from Korean fermented seasonings differed among Korean, UAE, and US consumers, and emphasized that consumer familiarity with the corresponding flavor should be considered in product development. In the present study, the cinnamon, ginger, and herbal attributes characterizing sample 825 were positioned away from the acceptability attributes in the MFA map. However, this pattern does not establish that these individual flavor notes reduced liking, because the samples differed in multiple sensory characteristics and familiarity with each flavor was not assessed. Further research should measure flavor-specific familiarity and directly test whether modifying these flavor notes affects consumer acceptance.

Overall, the MFA map showed that nutty flavor, rice syrup flavor, honey flavor, light-colored appearance, and soft texture were located on the same side of F1 as the acceptability attributes, whereas oil-related and spice-related attributes were generally located on the opposite side. Taken together, the MFA results suggest that taste- and aroma-related attributes were primarily associated with the differences in consumer responses among the yakgwa samples, with textural attributes providing an additional dimension of differentiation.

### 3.6. Limitations and Future Research

This study has several limitations. First, all participants were young Vietnamese women aged 18–29 years who were living or studying in Korea at the time of the assessment and were recruited from a single university in Seoul. Their exposure to Korean culture and food may have influenced their sensory perceptions and acceptance of yakgwa, and their responses may therefore differ from those of consumers residing in Vietnam. Familiarity and repeated exposure can increase the acceptance of unfamiliar foods [6,11]; however, the duration of residence in Korea and the extent of exposure to Korean food were not assessed in this study. Therefore, the magnitude and direction of these potential influences could not be determined. Nevertheless, 42 participants (40.4%) had never consumed yakgwa, and most participants with prior consumption experience reported consuming it rarely or only once or twice a year (Table 4). This suggests that exposure to Korean food culture did not necessarily imply familiarity with yakgwa. Limited product familiarity may also have influenced participants’ evaluations; however, food neophobia and expectations regarding Korean traditional foods were not measured. In addition, the four samples were selected to represent the range of product types available on the Korean market and may not cover the full range of sensory characteristics relevant to Vietnamese consumers. Accordingly, the findings should be interpreted within the context of this specific study sample and cannot be generalized to Vietnamese consumers as a whole, particularly those residing in Vietnam.

Second, the restriction of the sample to young women limits the applicability of the findings to male consumers and other age groups. Future studies should include consumers residing in Vietnam with broader representation across genders and age groups. For studies involving Vietnamese consumers living abroad, the duration of residence and the extent of exposure to Korean food culture should also be assessed. Measuring familiarity with yakgwa and with individual flavor notes, such as cinnamon flavor, ginger flavor, and herbal flavor, together with food neophobia, would help clarify how these factors relate to sensory perceptions and acceptance.

## 4. Conclusions

This study analyzed the sensory characteristics and consumer acceptability of four commercially available yakgwa products sold in Korea, targeting Vietnamese consumers. Additionally, it analyzed the relationships between sensory characteristics, consumer acceptability, and behavioral intentions. Four products were selected from 12 preliminary commercial samples to represent different product types, considering product popularity and online availability. A CATA evaluation using 39 sensory and image attributes was conducted with 104 Vietnamese consumers. In addition, acceptability, familiarity, willingness to eat again, and recommendation intention were rated on a 9-point scale. The data were analyzed using Cochran’s Q test, analysis of variance (ANOVA), correspondence analysis (CA), and multiple factor analysis (MFA). In the acceptability evaluation, samples 537 and 681 received significantly higher ratings than samples 214 and 825 for appearance, sweetness, texture, aftertaste, flavor, and overall acceptability, as well as for willingness to eat again, recommendation intention, and familiarity. These results indicate that samples 537 and 681 were more favorably evaluated by the Vietnamese consumers. In the CATA evaluation, sample 214 was characterized by oily-looking appearance, oily flavor, stale oily odor, crispy, and dry and rough texture, whereas sample 825 was characterized by dark-colored appearance, cinnamon aroma and flavor, ginger aroma and flavor, salty taste, and herbal flavor. In contrast, samples 537 and 681 were both characterized by light-colored appearance, sweet taste, and softness, with sample 537 additionally associated with honey aroma, nutty aroma, and weak aroma. A traditional snack-like image was selected with relatively high frequency for all four samples, suggesting that Vietnamese consumers perceive yakgwa as a traditional Korean food regardless of the sensory differences among products.

The CA results showed that the commercially available yakgwa samples were differentiated mainly by color, spice-related aromas, sweetness-related attributes, and texture. In the MFA, samples 214 and 825 were located in a different direction from the acceptability and behavioral intention attributes. Sample 537 was associated with nutty aroma, nutty flavor, rice syrup flavor, and light-colored, and was located in a similar direction to overall acceptability, familiarity, and recommendation intention, whereas sample 681 was associated with honey aroma, honey flavor, and a gift-worthy image, and was located close to the acceptability attributes of taste, texture, appearance, and aftertaste. As MFA describes relative associations among variables, however, these results should not be interpreted as indicating that specific sensory characteristics directly increased acceptability or behavioral intentions. Taken together, these findings suggest that responses to yakgwa within this study sample may reflect associations with multiple sensory and product-image attributes rather than a single characteristic. Nutty aroma, nutty flavor, rice syrup flavor, honey aroma, and soft and chewy textures may warrant further investigation as potential attributes of interest for product development. However, the findings do not establish whether modifying these attributes, or reducing oily flavor, stale oily odor, dry and rough texture, or pronounced spice-related flavors, would improve acceptance. Such recommendations require further validation among consumers residing in Vietnam, including an assessment of familiarity with individual flavors.

This study provides information on the relationship between the sensory characteristics of yakgwa, a traditional Korean dessert, and consumer responses among Vietnamese consumers residing in Korea. However, the sample consisted exclusively of young Vietnamese women recruited from a single university in Seoul, whose exposure to Korean food culture may have influenced their sensory perceptions and acceptance. In addition, a considerable proportion of participants had limited prior experience with yakgwa, which may also have influenced their evaluations. Therefore, the findings should be interpreted as exploratory and specific to the study sample. They cannot be generalized to Vietnamese consumers as a whole, particularly those living in Vietnam, or used to predict acceptance in the broader Vietnamese market. Further research involving consumers residing in Vietnam across a broader range of regions, genders, and age groups, and assessing familiarity with yakgwa and its specific flavor attributes, is needed to evaluate the applicability of these findings. Despite these limitations, this study provides preliminary insights into the sensory attributes associated with yakgwa acceptance among young Vietnamese women residing in Korea and offers a basis for further cross-cultural sensory research on traditional Korean desserts.

## Figures and Tables

**Table 1:** Product information and ingredient lists, including declared countries of origin, of the four commercial yakgwa samples.

Sample Code	Product Category	Ingredient List
214	Layered-style	Wheat flour (28%; wheat from the United States and Australia), rice syrup (made from 100% Korean rice), wheat flour (10%; Korean wheat), rapeseed oil (Canada), eggs, soju (ethyl alcohol), rice bran oil (Thailand), starch syrup (corn starch), corn flour, whole milk powder, cinnamon powder (2%; Vietnam), honey from sugar-fed bees (1.1%; Korea), refined salt, and baking powder
537	Commercially produced	Wheat flour (60%; wheat from Australia and the United States), starch syrup, sugar, corn oil (United States, Russia, Brazil, and other countries), sodium bicarbonate, and soybean oil (Argentina, the United States, Vietnam, and other countries)
681	Handmade	Wheat flour (35%), starch syrup (17%), corn oil (16.3%), glutinous rice (15%; Korea), sugar (10%), purified water (5%), refined salt (1%), cinnamon (0.5%), and sodium bicarbonate (0.2%).
825	Fusion-style	Rice syrup (46%; Korean rice, malt, and enzymes), wheat flour (wheat from the United States and Australia), canola oil (Canada, the United States, Australia, and other countries; silicone resin), glutinous rice flour (Korean glutinous rice), sugars (sugar and other sugars), liquid egg yolk, leavening preparation (sodium bicarbonate, sodium acid pyrophosphate, starch, monocalcium phosphate, and calcium lactate), jujube powder, salt, and cinnamon powder

**Table 2:** Purchase and label date information of the four commercial yakgwa samples for each evaluation session.

Sample Code	Product Category	Session ^(1)^	Purchase Date	Date on Label	Shelf Life ^(2)^
214	Layered-style	1	28 June 2026	Use-by: 17 June 2027	Not stated
		2	10 September 2026	Use-by: 10 October 2027	Not stated
537	Commercially produced	1	25 June 2026	Use-by: 30 January 2027	Not stated
		2	10 September 2026	Use-by: 14 August 2027	Not stated
681	Handmade	1	25 June 2026	Manufactured: 22 May 2026	4 months
		2	10 September 2026	Manufactured: 18 August 2026	4 months
825	Fusion-style	1	28 June 2026	Manufactured: 28 May 2026	6 months
		2	10 September 2026	Manufactured: 6 August 2026	6 months

^(1)^ Session 1, 2–10 July 2026; Session 2, 11–14 September 2026. ^(2)^ Counted from the date of manufacture.

**Table 3:** Label-declared nutritional composition per 100 g and percentages of daily reference values for the four commercial yakgwa samples.

Nutrient	214	537	681	825
Energy (kcal)	461.3 (23.1%)	441.6 (22.1%)	250.0 (12.5%)	425.0 (21.3%)
Carbohydrate (g)	63.3 (19.5%)	77.6 (24.0%)	49.0 (15.1%)	80.0 (24.7%)
Total sugars (g)	16.7 (16.7%)	25.6 (25.6%)	16.0 (16.0%)	15.0 (15.0%)
Total fat (g)	20.0 (37.0%)	12.8 (23.7%)	5.0 (9.3%)	10.8 (19.9%)
Saturated fat (g)	3.3 (22.2%)	1.6 (10.7%)	1.0 (6.7%)	1.5 (10.0%)
Trans fat (g)	0 (—)	0 (—)	0 (—)	0 (—)
Cholesterol (mg)	0 (0%)	0 (0%)	0 (0%)	37.5 (12.5%)
Protein (g)	6.7 (12.1%)	4.0 (7.3%)	3.0 (5.5%)	2.5 (4.5%)
Sodium (mg)	13.3 (0.7%)	52.0 (2.6%)	120.0 (6.0%)	400.0 (20.0%)

Values are expressed as amounts per 100 g, with percentages of Korean daily reference values in parentheses: carbohydrate, 324 g; total sugars, 100 g; total fat, 54 g; saturated fat, 15 g; cholesterol, 300 mg; protein, 55 g; and sodium, 2000 mg. Energy percentages are based on 2000 kcal/day. Percentages were calculated before rounding. —: no established daily reference value. Values were derived from product labels rather than measured in this study.

**Table 4:** Yakgwa consumption characteristics of the Vietnamese participants (*n* = 104).

Variables	Categories	*n*	%
Sex	Female	104	100.0
Age (years)	18–19 20–24 25–29	23 67 14	22.1 64.4 13.5
Age (Mean ± SD)		21.85 ± 2.55	
Experience of Yakgwa consumption	Yes No	62 42	59.6 40.4
Frequency of Yakgwa consumption	Rarely 1–2 times/year Less than once/month 1–3 times/month At least once/week	18 35 6 2 1	29.0 56.5 9.7 3.2 1.6
Experience of Yakgwa purchase	Yes No	37 25	59.7 40.3
Preference for Yakgwa (Mean ± SD)		3.24 ± 0.79	
Interest in Korean traditional desserts (Mean ± SD)		3.62 ± 0.84	

Preference for yakgwa was measured on a 5-point scale (1 = do not like at all, 3 = neutral, 5 = like very much), and interest in traditional Korean desserts was measured on a 5-point scale (1 = not at all interested, 3 = neutral, 5 = very interested).

**Table 5:** Purpose of Yakgwa Consumption or Purchase and Factors Considered When Purchasing Yakgwa.

Variables	Categories	*n*	%
Purpose of Yakgwa consumption or purchase	As a snack To experience traditional food As a gift For holidays or ancestral rites Other To try a trendy dessert To experience new products	33 32 21 10 6 3 2	48.5 47.1 30.9 14.7 8.8 4.4 2.9
Factors considered when purchasing Yakgwa	Taste Price Texture Healthiness Package design Brand Ingredients	56 20 16 15 10 6 6	82.4 29.4 23.5 22.1 14.7 8.8 8.8

Multiple responses were allowed. Percentages were calculated based on 68 respondents and may sum to more than 100%.

**Table 6:** Consumer Acceptability of Four Commercial Yakgwa Samples evaluated by Vietnamese consumers (*n* = 104).

	214		537		681		825		*p*-Value
	Mean	SD	Mean	SD	Mean	SD	Mean	SD	
Appearance	5.93 ^b^	1.69	6.80 ^a^	1.57	6.96 ^a^	1.48	5.81 ^b^	1.60	<0.001
Color	6.30 ^b^	1.83	6.68 ^ab^	1.70	6.96 ^a^	1.44	5.77 ^c^	1.89	<0.001
Aroma	6.20 ^ab^	1.77	6.49 ^a^	1.81	6.62 ^a^	1.67	5.99 ^b^	1.71	0.040
Sweetness	5.91 ^b^	1.89	6.50 ^a^	1.68	6.43 ^a^	1.68	5.74 ^b^	1.90	0.003
Nutty flavor	6.05 ^ab^	1.84	6.40 ^a^	1.72	6.48 ^a^	1.55	5.78 ^b^	1.83	0.013
Oiliness	5.69	2.11	6.12	1.86	6.18	1.77	5.63	1.83	0.071
Texture	5.82 ^b^	1.79	6.73 ^a^	1.82	6.67 ^a^	1.69	5.96 ^b^	1.73	<0.001
Aftertaste	5.77 ^b^	1.81	6.73 ^a^	1.73	6.53 ^a^	1.63	5.78 ^b^	1.80	<0.001
Flavor	5.89 ^b^	1.77	6.40 ^a^	1.58	6.59 ^a^	1.54	5.95 ^b^	1.70	<0.001
Overall acceptability	6.10 ^b^	1.80	6.58 ^a^	1.39	6.82 ^a^	1.44	5.99 ^b^	1.70	<0.001
Willingness to eat again	5.69 ^b^	2.06	6.77 ^a^	1.63	6.82 ^a^	1.40	5.62 ^b^	2.06	<0.001
Recommendation intention	5.63 ^b^	2.00	6.57 ^a^	1.54	6.64 ^a^	1.40	5.60 ^b^	1.91	<0.001
Familiarity	5.91 ^b^	2.04	6.80 ^a^	1.57	6.74 ^a^	1.46	5.74 ^b^	1.94	<0.001

9-point hedonic scale: 1 = Dislike extremely, 5 = Neither like nor dislike, 9 = Like extremely; *p*-value obtained through one-way analysis of variance; ^a–c^ Different superscript letters within a row indicate significant differences among the samples at *p* < 0.05 by Duncan’s multiple range test.

**Table 7:** Sensory Characteristics of Four Commercial Yakgwa Samples Based on the Check-All-That-Apply (CATA) Method.

		214		537		681		825		*p*-Value
		n	%	n	%	n	%	n	%	
Appearance	Light-colored	26 ^b^	25.00	62 ^a^	59.62	64 ^a^	61.54	11 ^b^	10.58	<0.001
	Dark-colored	36 ^b^	34.62	20 ^bc^	19.23	12 ^c^	11.54	84 ^a^	80.77	<0.001
	Glossy	30 ^ab^	28.85	18 ^bc^	17.31	34 ^a^	32.69	14 ^c^	13.46	<0.001
	Oily-looking	47 ^a^	45.19	15 ^b^	14.42	25 ^b^	24.04	13 ^b^	12.50	<0.001
	Dry-looking	17 ^ab^	16.35	21 ^a^	20.19	7 ^b^	6.73	18 ^ab^	17.31	0.037
Aroma	Honey aroma	19 ^b^	18.27	36 ^a^	34.62	26 ^ab^	25.00	25 ^ab^	24.04	0.036
	Rice syrup aroma	10 ^a^	9.62	15 ^a^	14.42	18 ^a^	17.31	13 ^a^	12.50	0.356
	Nutty aroma	25 ^ab^	24.04	36 ^a^	34.62	27 ^ab^	25.96	19 ^b^	18.27	0.040
	Stale oily odor	33 ^a^	31.73	20 ^a^	19.23	20 ^a^	19.23	22 ^a^	21.15	0.108
	Weak aroma	14 ^b^	13.46	30 ^a^	28.85	22 ^ab^	21.15	10 ^b^	9.62	<0.001
	Ginger aroma	13 ^ab^	12.50	4 ^b^	3.85	14 ^ab^	13.46	18 ^a^	17.31	0.016
	Cinnamon aroma	40 ^a^	38.46	6 ^b^	5.77	27 ^a^	25.96	34 ^a^	32.69	<0.001
Taste /Flavor	Sweet taste	32 ^bc^	30.77	56 ^a^	53.85	49 ^ab^	47.12	31 ^c^	29.81	<0.001
	Excessively sweet taste	4 ^b^	3.85	4 ^b^	3.85	9 ^ab^	8.65	17 ^a^	16.35	<0.001
	Wheat flour flavor	20 ^a^	19.23	21 ^a^	20.19	13 ^a^	12.50	17 ^a^	16.35	0.380
	Ginger flavor	10 ^ab^	9.62	1 ^b^	0.96	11 ^a^	10.58	12 ^a^	11.54	0.008
	Honey flavor	19 ^a^	18.27	20 ^a^	19.23	18 ^a^	17.31	16 ^a^	15.38	0.881
	Rice syrup flavor	11 ^a^	10.58	12 ^a^	11.54	10 ^a^	9.62	6 ^a^	5.77	0.473
	Nutty flavor	37 ^a^	35.58	30 ^ab^	28.85	21 ^bc^	20.19	14 ^c^	13.46	<0.001
	Oily flavor	40 ^a^	38.46	28 ^ab^	26.92	31 ^ab^	29.81	21 ^b^	20.19	0.027
	Rich flavor	7 ^a^	6.73	8 ^a^	7.69	15 ^a^	14.42	14 ^a^	13.46	0.166
	Cinnamon flavor	24 ^a^	23.08	4 ^b^	3.85	20 ^a^	19.23	25 ^a^	24.04	<0.001
	Salty taste	6 ^b^	5.77	1 ^b^	0.96	10 ^b^	9.62	25 ^a^	24.04	<0.001
	Herbal flavor	4 ^b^	3.85	4 ^b^	3.85	5 ^b^	4.81	21 ^a^	20.19	<0.001
Texture	Crispy	24 ^a^	23.08	3 ^b^	2.88	3 ^b^	2.88	2 ^b^	1.92	<0.001
	Soggy	10 ^a^	9.62	10 ^a^	9.62	12 ^a^	11.54	15 ^a^	14.42	0.590
	Hard	19 ^a^	18.27	2 ^b^	1.92	11 ^ab^	10.58	6 ^b^	5.77	<0.001
	Soft	28 ^b^	26.92	64 ^a^	61.54	48 ^a^	46.15	55 ^a^	52.88	<0.001
	Moist	12 ^a^	11.54	22 ^a^	21.15	20 ^a^	19.23	26 ^a^	25.00	0.062
	Chewy	8 ^b^	7.69	20 ^a^	19.23	20 ^a^	19.23	11 ^ab^	10.58	0.011
	Dense	10 ^a^	9.62	4 ^a^	3.85	12 ^a^	11.54	7 ^a^	6.73	0.142
	Sticks to teeth	17 ^a^	16.35	7 ^ab^	6.73	10 ^ab^	9.62	6 ^b^	5.77	0.028
	Dry and rough	35 ^a^	33.65	6 ^b^	5.77	14 ^b^	13.46	15 ^b^	14.42	<0.001
	Light mouthfeel	12 ^b^	11.54	27 ^a^	25.96	21 ^ab^	20.19	19 ^ab^	18.27	0.036
Image	Traditional snack-like	65 ^a^	62.50	55 ^a^	52.88	60 ^a^	57.69	55 ^a^	52.88	0.308
	Premium dessert-like	8 ^a^	7.69	12 ^a^	11.54	13 ^a^	12.50	16 ^a^	15.38	0.282
	Trendy	6 ^a^	5.77	10 ^a^	9.62	8 ^a^	7.69	6 ^a^	5.77	0.648
	Familiar	34 ^a^	32.69	34 ^a^	32.69	32 ^a^	30.77	29 ^a^	27.88	0.813
	Gift-worthy	12 ^b^	11.54	21 ^ab^	20.19	28 ^a^	26.92	16 ^ab^	15.38	0.016

Values are expressed as the number of consumers selecting each attribute (n) and the corresponding percentage (%) of the 104 participants. Different superscript letters within the same row indicate significant differences among samples (*p* < 0.05); the letter a denotes the highest value. *p*-values were obtained using Cochran’s Q test.

## Data Availability

The original contributions presented in the study are included in the article; further inquiries can be directed to the corresponding author.
